# Patient-reported outcomes with a personalized follow-up program after lung cancer resection: A single-center randomized controlled trial

**DOI:** 10.1016/j.apjon.2025.100844

**Published:** 2026-01-02

**Authors:** Yiqing Luo, Yuna Cheng, Zuodong Song, Hui Chen, Yinping Bo, Haobo Shixing

**Affiliations:** a Department of Oncology Surgery, Shanghai Chest Hospital, Shanghai Jiao Tong University School of Medicine, Shanghai, China; b Department of Thoracic Surgery, Shanghai Chest Hospital, Shanghai Jiao Tong University School of Medicine, Shanghai, China; c Department of Intensive Care Unit, Shanghai Chest Hospital, Shanghai Jiao Tong University School of Medicine, Shanghai, China

**Keywords:** Lung neoplasm, Postoperative, Patient-reported outcome, Follow-up, Quality of life, Self-management efficacy

## Abstract

**Objective:**

This study aims to determine the impact of PROM with a personalized follow-up program on the evaluation of quality of life and self-management for patients after lung cancer resection.

**Methods:**

Given a formal power calculation a total of 240 patients with lung cancer. Participants were randomly assigned to either the experimental or the control groups. Patients in the experimental group received a personalized follow-up program of patient-reported outcomes. The control group received only the telephone follow-up. Baseline data (T0) were collected before the intervention (on the day of discharge), and quality of life, self-efficacy, and compliance were measured at 2 weeks (T1), 4 weeks (T2), and 4 months (T3) post-discharge.

**Results:**

The difference in quality of life between the experimental and control groups was significant (Wald χ^2^ = 5.204, *P* = 0.023), with the experimental group showing significantly better quality of life at T2 compared to the control group (*t* = 2.515, *P* = 0.013). Both groups showed improvements in quality of life at all post-test time points (Wald χ^2^ = 574.167, *P* < 0.001), and the interaction between group and time was not statistically significant (Wald χ^2^ = 2.354, *P* = 0.308). Regarding self-management efficacy, Generalized Estimating Equations results indicated a significant difference between the experimental and control groups (Wald χ^2^ = 6.573, *P* = 0.010), with the experimental group showing significantly higher self-management efficacy at T2 and T3 compared to the control group (*t* = 3.024, *P* = 0.003; *t* = 2.214, *P* = 0.028). No significant differences were observed at T0 and T1. Both groups showed improvements in self-management efficacy at all post-test time points (Wald χ^2^ = 301.390, *P* < 0.001), and the interaction between group and time was not statistically significant (Wald χ^2^ = 3.971, *P* = 0.137).

**Conclusions:**

For patients after lung cancer surgery, the program has optimized the evaluation of postoperative quality of life and self-management efficacy.

**Trial registration:**

Chinese Clinical Trial Registry (NCT06483295).

## Introduction

Lung cancer is the most prevalent type of cancer globally, with both the incidence and mortality rates ranking highest among all cancers.^1^ Surgical resection remains the preferred curative treatment for lung cancer. The Enhanced Recovery After Surgery (ERAS) pathway reduces postoperative hospital stay for lung cancer patients; however, it also results in patients often being discharged during the early or middle stages of recovery rather than the late stages.^2^^,^^3^ Invasive surgical trauma^2^, ^3^, ^4^ leads to a significant decline in exercise tolerance within the first-week post-surgery, lung function and physical performance within two weeks, and daily activity levels within four weeks compared to preoperative levels.^5^, ^6^, ^7^, ^8^ The incidence of various symptoms ranges from 48% to 79%,^9^^,^^10^ and it can take 1–4 months or longer to return to preoperative normal activity levels,^11^, ^12^, ^13^ profoundly impacting quality of life.^2^ Therefore, postoperative continuity of care is crucial, challenging the medical team's ability to make efficient and precise diagnostic and intervention decisions within a limited time frame.^14^

The widespread use of smartphones has facilitated the emergence of follow-up methods based on patient-reported data.^15^ These methods enable immediate and convenient remote monitoring of postoperative symptoms, providing a solid foundation for dynamically adjusting treatment strategies^16^ and accelerating patient recovery.^17^ Although the studies conducted by Tang et al.^18^ and Dai et al.^19^ have revealed the potential of patient-reported outcome-based information technology in reducing the symptom burden of patients, indicating that it can achieve efficient and regular symptom assessment in a busy clinical environment, thereby bringing substantial health benefits to patients,^20^ the existing evidence has key limitations.

Currently, related studies primarily employ longitudinal designs to evaluate the feasibility^18^ and acceptability^18^ of the tools, survival rates,^21^ compliance,^22^ symptom trajectory changes,^22^ cost-effectiveness,^23^ and tumor recurrence monitoring.^24^ Most studies adopted non-comparative^20^ or retrospective data.^21^ Only three randomized controlled trials could be identified by the authors,^19^^,^^20^^,^^25^ and the actual impact on patients' quality of life and self-management efficacy remains unclear, failing to provide a comprehensive view of the effectiveness of continuity of care in postoperative patients. One clinical trial only used patient-reported outcomes (PROs) to assess the incidence of symptom threshold events.^20^ This could affect the comprehensiveness and generalizability of the results, offering limited guidance for clinical practice and research.

Information technology based on PROs holds promise as a key strategy for identifying and managing actionable symptoms, thereby optimizing the overall health of lung cancer patients. It also reminds us that future research should be dedicated to building a more comprehensive and extensive patient reporting framework to fully test the actual effectiveness of mobile health technology in multi-symptom and multi-dimensional assessment.^26^ To this end, our research team developed a personalized follow-up program for lung cancer patients based on PROs using a WeChat mini-program. This program enables nurse-led teams to conduct online and offline interactive interventions. We initially investigated its effects on the quality of life and self-management efficacy of lung cancer patients at 2 weeks, 4 weeks, and 4 months post-surgery, as well as the experimental group's compliance and perceptions of the system. This was accomplished through a randomized controlled trial.

## Methods

### Study design and setting

This study is a single-center, prospective, single-blind (assessor-blinded), randomized controlled trial. The trial was registered with the Chinese Clinical Trial Registry (Registration No. NCT06483295) and received ethical approval from the Institutional Review Board of Shanghai Chest Hospital (Approval No. IS22099). All participants provided written informed consent.

### Participants

Recruitment was conducted in the Fourth Ward of the Oncology Surgery Department at Shanghai Chest Hospital, which performs over 12,000 thoracic surgeries annually. This ward has 51 beds and an average of over 2700 lung cancer surgeries per year. Participant recruitment began on 14 March 2024, with the study commencing on 13 March 2024. All research plans and data collection were completed on 7 July 2024, following the three-month follow-up of the last discharged patient.

The inclusion criteria for patients were: (1) Diagnosed with non-small cell lung cancer without distant metastasis or other tumors and undergoing surgery with curative intent; (2) Aged 18 years or older; (3) Informed about their disease diagnosis and treatment; (4) Able to express themselves; and (5) Able to read and write using a smartphone. The exclusion criteria for patients were: (1) Cognitive impairments (understanding or expressing themselves) (2) History of being intolerant to surgery (e.g., hemodynamic instability requiring an Intensive Care Unit [ICU] stay of > 48 hours, refractory hypoxemia, or other life-threatening complications that preclude continued participation in the study); or having severe complications (Clavien-Dindo grade ≥ III); (3) Presence of psychiatric disorders or cognitive impairments; and (4) Participation in another intervention study.

### Sample size

The sample size was determined a priori for the primary outcome of quality of life score, using a two-independent-sample comparison of means. Based on the post-intervention scores reported by Sommer et al.^27^ (control group: 102.0 ± 21.2; intervention group: 110.2 ± 15.6), the effect size was calculated as Cohen's d = 0.44, which was used for the sample size estimation. The calculation was performed with PASS 15.0 software, with the parameters set as two-sided *α* = 0.05 and statistical power = 0.8 (i.e., 1−β = 0.8, *β* = 0.2). Under these parameters, approximately 81 participants per group were required. To account for an estimated 20% attrition rate, the recruitment target was adjusted to 102 participants per group, yielding a total of 204 participants. Ultimately, 240 participants were enrolled in the study, which exceeds the planned sample size and ensures sufficient statistical power for the primary comparison.

### Randomization, concealment, and blinding

Research Assistant A assessed the eligibility of participants, obtained written informed consent, and collected baseline data. Research Assistant B, who was not involved in the recruitment process, performed the random allocation using a random sequence set generated from the research randomizer website (https://www.randomizer.org/). The numeric sequence consisted of 240 unique numbers. Research Assistant A randomly assigned consenting participants who completed the baseline assessment to either the experimental or control groups in a 1:1 ratio, based on their enrollment order and the random codes on the envelopes. Due to the nature of the interventions in this study, blinding of the participants and the nursing researchers (QW) implementing the interventions was not possible. To minimize potential performance bias, the nursing team received standardized training emphasizing strict adherence to group-specific protocols and standardized scripts. Additionally, workflow segregation was implemented for the intervention nurses, with different shifts assigned to deliver the experimental and control interventions. To ensure protocol fidelity, 10% of follow-up recordings were randomly selected periodically for compliance auditing. However, the recruiter (Research Assistant A) was unaware of the group assignments.

### Intervention

Firstly, we conducted a scoping review to clarify the use of mobile information platforms based on PROs in studies involving discharged lung cancer patients. This review encompassed patient-reported content, PROs tools (including threshold settings), and data collection timing. We found only two studies utilizing randomized controlled trials, with one focusing exclusively on advanced lung cancer patients. Subsequently, we conducted semi-structured interviews with 27 stakeholders, including 9 thoracic surgery health care providers (2 doctors and 7 nurses), and 9 lung cancer patients and caregivers, to identify implementation facilitators and barriers.

Based on the evidence and stakeholder inputs, we drafted an initial plan and invited feedback from 16 experts, including surgeons, clinical nursing experts, nursing managers, nursing educators, and evidence-based methodologists. The final plan was revised and refined according to their feedback. We identified four implementation strategies for personalized follow-up projects for lung cancer patients based on patient-reported outcomes: evidence transformation, multidisciplinary cooperation, team formation and training, and patient-reported outcome tool settings (application ports and functions). Detailed intervention content is provided in Appendix A.

In this project, we selected and integrated the MD Anderson Symptom Inventory (MDASI)^28^ as the built-in patient-reported outcome tool within the mini-program. Additionally, we incorporated a lung cancer-specific assessment module to provide more precise health monitoring services. However, the lung cancer assessment module developed by the MD Anderson Symptom Center focuses on only three symptoms: cough, constipation, and sore throat. Based on past research data and our team's extensive clinical experience, we found that these indicators do not fully cover the typical symptom manifestations of lung cancer patients in China. Therefore, to better meet the actual needs of Chinese patients, we combined the lung cancer-specific symptom module designed by Wang et al.^29^ with the MD Anderson Symptom Inventory, resulting in a more comprehensive and targeted assessment system.

In the WeChat mini-program-based lung cancer follow-up project, patients first register and log into the WeChat mini-program. They receive regular reminders to complete the MD Anderson Symptom Inventory (MDASI) and submit their reports. Patients are prompted to report twice weekly for the first month post-discharge, then once weekly until 4 months post-discharge, or as needed based on their symptom perception. Surveys are sent three times a day at each time point until the patient responds.^22^ Patients can view historical records, access health education content, provide feedback on their usage, and consult for any questions they might have. Health care providers log into the WeChat mini-program to review the symptom reports submitted by patients, paying particular attention to reports exceeding threshold values. They record and implement intervention measures as necessary. Additionally, health care providers manage patient information and report data, conduct statistical analyses and trend analysis, and communicate with patients to answer their questions and provide recommendations. Based on individualized symptom assessment, nurses implemented personalized education plans for each participant according to the patient-reported data. Upon completion of the 4-month intervention period, the system's core functionalities remained accessible for voluntary patient use; however, active monitoring and responsive support from the specialized clinical team were systematically discontinued (Table 1, Appendix A and B).

**Table 1:** Core components of the PRO-based personalized follow-up program.

Core component	Specifications and procedures
1. Symptom assessment	**Tool:** MD Anderson Symptom Inventory (MDASI) combined with a lung cancer-specific module
	**Frequency:** Twice weekly in the first month post-discharge, then once weekly until 4 months postoperatively
	**Scoring:** 1–10 (Severity and interference)
2. Alert thresholds & triggers	**Yellow alert:** Single item score 4–5
	**Red alert:** Single item score ≥ 6
3. Response timeframes	**Yellow alert:** Specialist nurse telephone assessment and first-level intervention within 24 hours
	**Red alert:** Specialist nurse urgent assessment within 4 hours with immediate attending physician notification via the system
4. Tiered intervention & decision pathways	**Routine content delivery:** • Video tutorials via WeChat mini-program (Covering breathing regulation, relaxation training, and other evidence-based self-management skills) • Graphic materials (Covering scientific knowledge of symptoms, energy conservation, dietary optimization, posture guidance, personalized exercise plans, and symptom-specific coping strategies) **First-level (Nurse-led):** • Non-pharmacological advice (e.g., breathing techniques, dietary adjustments) based on the symptom management resource library • Health education and psychological support **Second-level (Physician-led):** • Medication adjustment, earlier outpatient appointment, or emergency referral based on clinical assessment
5. Multidisciplinary coordination	**Regular meetings:** Weekly Multidisciplinary Team (MDT) meetings every Friday afternoon to review all alert cases
	**Consultation on demand:** System-enabled referrals to nutrition, psychology, or rehabilitation teams as needed
6. System support & Automation	**Patient terminal:** Automated reminders (14:00 on reporting days), auto-save function, historical records with trend graphs
	**Staff terminal:** Abnormal report list, internal messaging system, standardized response templates, data statistics

PRO, patient-reported outcome; SD, standard deviation.

### Usual care

Participants in the control group received standard care including brief guidance from thoracic surgery nurses. The main components of the guidance were: (1) Breathing Function Training: Continue daily breathing exercises after discharge. (2) Exercise Rehabilitation Training: Perform daily arm-lifting exercises on the surgical side and engage in moderate aerobic activities such as walking and Tai Chi. Gradually increase activity levels to avoid fatigue. Refrain from lifting heavy objects or engaging in strenuous physical labor within 4 months post-surgery. (3) Monitoring and Self-Management of Common Postoperative Symptoms: Monitor for symptoms such as elevated temperature, dry cough, and pain. (4) Wound Care: Keep the wound clean and dry. (5) Lifestyle Adjustments: Abstain from smoking and alcohol. Follow a diet high in calories, protein, and vitamins, but low in salt and fat. Avoid spicy foods, coffee, and strong tea. Follow-up instructions for the first month after discharge were also provided.

Standard care was provided by the same nursing team as the intervention group. Additionally, participants received a monthly phone call for 4 months and an educational booklet during their outpatient follow-up at the end of the study (four months post-surgery) to adhere to research ethics.

### Measures

#### Personal information questionnaire

The evaluation utilized a self-designed questionnaire to gather comprehensive patient information and basic disease data. General information encompassed patient demographics such as gender, age, BMI, education level, marital and employment status, place of residence, and monthly household income per capita. Basic disease details included whether patients underwent postoperative chemotherapy or radiotherapy, tumor type, surgical approach, type of surgery, preoperative lung function, comorbid chronic diseases, duration of chest tube placement, postoperative hospital stay, and total hospitalization duration. Data were retrieved from patients' medical records.

#### Quality of life measurements

Functional Assessment of Cancer Therapy Lung Cancer, FACT-L Developed by Cella et al.^30^ and translated into Chinese by Wan et al.,^31^ this scale comprises a general module for assessing cancer therapy's functional impact and a lung cancer-specific module tailored to evaluate the quality of life among lung cancer patients. The scale covers five dimensions: physical well-being, social/family well-being, functional well-being, emotional well-being, and additional concerns, totaling 36 items. Responses are recorded on a five-point Likert scale, with positively worded items ranging from 0 (not at all) to 4 (very much), and negatively worded items reverse-scored. Scores range from 0 to 144, with higher scores indicating better quality of life. The scale's internal consistency, measured by Cronbach's α coefficient, was validated at 0.805.

#### Self-management efficacy measurements

Strategies Used by People to Promote Health, SUPPH Developed by Lev et al.^32^ and translated into Chinese by Qian et al.,^33^ this scale demonstrates robust reliability and validity. It comprises three dimensions: self-decompression, self-decision making, and positive attitude, encompassing a total of 28 items. Responses are recorded on a 5-point Likert scale, ranging from “not confident at all” to “very confident,” scored from 1 to 5. The total score, ranging from 28 to 140, reflects the overall confidence level of postoperative lung cancer patients in managing their disease, with higher scores indicating greater confidence. The scale exhibits a Cronbach's *α* coefficient of 0.970, with coefficients for each dimension ranging from 0.849 to 0.959.

### Data collection

Data collection was performed by a research assistant who remained blinded to group assignments throughout the study. Following written informed consent, patients received explanations regarding the study's objectives and questionnaire completion requirements. On the day of discharge (T0), patients were instructed to complete paper-based quality of life and self-management efficacy questionnaires. At 2 weeks post-discharge (T1), data from the control group were gathered via telephone interviews, while the experimental group completed system-distributed questionnaires. To ensure questionnaire quality, data at 4 weeks (T2) and 4 months (T3) post-discharge were collected through paper-based questionnaires during outpatient visits. For patients missing appointments, telephone interviews were conducted by the research assistant to assess self-efficacy and quality of life. Patients were reminded of upcoming follow-ups after each session. To reduce potential reported bias in the telephone interview completely followed standardized script protocols and were uniformly trained by researchers to ensure operational consistency. Ideally, patients completed questionnaires independently; during telephone interviews, questions were read aloud and answers objectively recorded. Any missing, incomplete, or erroneous data were promptly addressed and corrected by the research assistant following questionnaire completion.

### Statistical analysis

The normality of distribution for all continuous variables, including quality of life and self-management efficacy scores, was formally assessed using the Shapiro–Wilk test. The results indicated that the data for both primary outcomes did not significantly deviate from normality (Quality of Life: *W* = 0.985, *P* = 0.127; Self-Management Efficacy: *W* = 0.978, *P* = 0.064). The *t*-test is used for continuous variables that are consistent with the normal distribution. Continuous variables following a normal distribution were analyzed using the *t*-test. Demographic and clinical characteristics between the two groups were compared using the Chi-square test or Fisher's exact test for categorical variables, and the Mann–Whitney *U* test for ordinal data. Generalized Estimating Equations (GEE) were employed to assess group, time, and interaction effects on quality of life and self-efficacy. Longitudinal data were analyzed using GEE, which provide valid statistical inference under the missing-at-random (MAR) assumption. This approach utilizes all available data points at each measurement timepoint without requiring data imputation or exclusion of cases with incomplete follow-up. The analysis included all 240 randomized participants (intention-to-treat population), with the model incorporating observed data from all available timepoints for each participant. A linearized approach with an unstructured correlation matrix was utilized in the GEE analysis, and post hoc tests were conducted using the least significant difference method. To control the risk of Type I error due to multiple comparisons, the Benjamini-Hochberg procedure was applied to correct the false discovery rate (FDR) at 5% for between-group comparisons across the four time points. Statistical analyses were conducted using SPSS 26.0 software, with significance set at *P* < 0.05 (two-tailed).

## Results

### Paticipants flow and recruitment

**Fig. 1:**
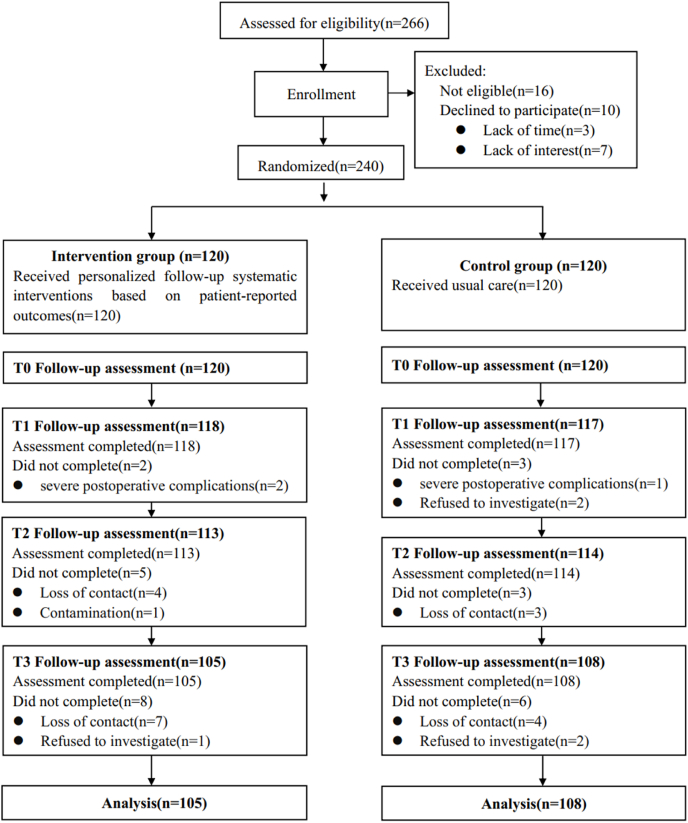
Consort flow diagram.

A total of 266 patients were initially enrolled in the study. Of these, 240 eligible participants who met the study criteria and provided voluntary consent were randomized into either the experimental group (*n* = 120) or the control group (*n* = 120). Of these, 213 participants (88.8%) completed the three-month follow-up: 105 in the experimental group and 108 in the control group. Reasons for withdrawal from the study included severe postoperative complications (3), loss of contact (18), refusal to follow-up (5), and contamination (1), This incident involved a participant in the experimental group who shared details about the WeChat mini-program with a control group participant whom they had met during their hospital stay) (Fig. 1).

Among the 213 subjects, the average age was 57.71 years, with 54.0% aged 60 years or older. The majority were female (59.6%) and had attained a high school education or higher (76.1%). Most were married (90.6%), employed (67.1%), and resided in urban areas (60.6%). A small proportion (2.3%) had a monthly household income per capita of less than 2000 yuan. Medically, the cohort predominantly had adenocarcinoma (96.2%), lacked chronic diseases (66.7%), and underwent minimally invasive thoracoscopic surgery (94.4%). Approximately 28.2% received postoperative radiotherapy or chemotherapy. During hospitalization, the average duration of chest tube placement was 64.84 hours, with an average postoperative hospital stay of 3.31 days and a total hospital stay averaging 6.19 days. Table 2 shows the Baseline Characteristics of Participants at Enrollment.

**Table 2:** Baseline characteristics of participants at enrollment.

Variable	Mean±SD or *n* (%)		*t/*χ^*2*^*/z*	*P*
	Intervention group (*n* = 105)	Control group (*n* = 108)		
**Age (years)**	57.55 ± 13.18	57.87 ± 11.29	−0.189	0.850^a^
**Sex**				
Male	42 (40.0)	44 (40.7)	0.012	0.912^b^
Female	63 (60.0)	64 (59.3)		
**Body mass index(kg/m^2^)**	23.69 ± 3.61	23.58 ± 2.65	0.242	0.809^a^
**Education level**				
Junior high school and below	25 (23.8)	26 (24.1)	−0.071	0.944^c^
High school or technical secondary school	12 (11.4)	8 (7.4)		
Junior college or undergraduate degree	59 (56.2)	68 (63.0)		
Master degree and above	9 (8.6)	6 (5.6)		
**Marital status**				
Unmarried	5 (4.8)	2 (1.9)	–	0.477^d^
Married	93 (88.6)	100 (92.6)		
Widowed or divorced	7 (6.7)	6 (5.6)		
**Work status**				
On job	72 (68.6)	71 (65.7)	1.456	0.483^b^
Retired	24 (22.9)	31 (28.7)		
Unemployed	9 (8.6)	6 (5.6)		
**Residence**				
Town	63 (60.0)	66 (61.1)	1.816	0.178^b^
Countryside	42 (40.0)	42 (38.9)		
**Monthly income per person (CNY)**				
≤ 2000	2 (1.9)	3 (2.8)	−0.933	0.351^c^
2001-4000	40 (38.1)	42 (38.9)		
4001-6000	28 (26.7)	37 (34.3)		
> 6000	35 (33.3)	26 (24.1)		
**Postoperative radiotherapy or chemotherapy**				
Yes	77 (73.3)	76 (70.4)	0.231	0.631^b^
No	28 (26.7)	32 (29.6)		
**Tumor type**				
Adenocarcinoma	100 (95.2)	105 (97.2)	–	0.494^d^
Others	5 (4.8)	3 (2.8)		
**Surgery type**				
Minimally invasive thoracoscopy	100 (95.2)	101 (93.5)	0.296	0.586^b^
Open surgery	5 (4.8)	7 (6.5)		
**Surgical methods**				
Wedge resection	33 (31.4)	31 (28.7)	2.984	0.394^b^
Segmentectomy	30 (28.6)	40 (37.0)		
Lobectomy	32 (30.5)	24 (22.2)		
Others	10 (9.5)	13 (12.0)		
**Comorbid chronic diseases**				
Yes	33 (31.4)	38 (35.2)	0.338	0.561^b^
Chronic obstructive pulmonary disease	15 (14.3)	18 (16.7)	0.247	0.619^b^
Cardiovascular diseases	12 (11.4)	14 (13.0)	0.125	0.724^b^
Diabetes mellitus	8 (7.6)	10 (9.3)	0.202	0.653^b^
No	72 (68.6)	70 (64.8)		
**Tobacco use**				
Smoker	44 (41.9)	45 (41.7)	0.001	0.970^b^
Non-smoker	61 (58.1)	63 (58.3)		
Mean pack-years (among smokers)	32.8 ± 18.9	32.2 ± 18.3	0.251	0.802^a^
**Preoperative lung function**				
FVC	2.94 ± 0.68	3.34 ± 2.93	−1.391	0.171^a^
FEV1/FVC	80.66 ± 6.91	82.25 ± 6.25	−1.766	0.079^a^
Chest drainage tube indwelling time (hours)	60.69 ± 36.92	68.88 ± 42.82	−1.494	0.136^a^
Postoperative hospital stay (days)	3.12 ± 1.80	3.49 ± 1.94	−1.429	0.154^a^
Total length of stay (days)	6.02 ± 2.40	6.36 ± 2.73	−0.971	0.333^a^

SD, standard deviation; FEV1, forced expiratory volume in 1 second; FVC; forced vital capacity.

a Independent samples *t*-test.

b Chi-square tests.

c Mann–Whitney *U* test.

d Fisher exact test.

### Effects on quality of life

**Fig. 2:**
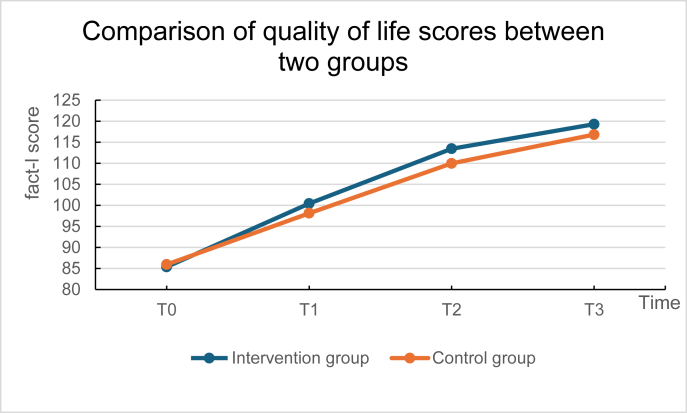
Changes in mean scores of quality of life.

GEE analysis revealed a significant main effect of group (Wald χ^2^ = 5.204, *P* = 0.023) and time (Wald χ^2^ = 574.167, *P* < 0.001) on quality of life; however, the group × time interaction was not statistically significant (*P* = 0.308). Post hoc between-group comparisons with multiple-comparison adjustment indicated that the intervention group demonstrated significantly better quality of life scores than the control group at the 1-month postoperative assessment (mean difference = 3.49, 95% CI [0.75, 6.23]), although the adjusted *P*-value was marginally significant (*P* = 0.052), suggesting a potential short-term benefit of the intervention. No statistically significant between-group differences were observed at other time points, including the day of discharge, 2 weeks, or 4 months postoperatively. As illustrated in Fig. 2, the intervention group exhibited a more pronounced improvement trend in quality of life scores from T0 to T3 compared to the control group (Table 3, Table 4, Table 5 and Fig. 2).

**Table 3:** Comparison of outcome variables of participants between intervention and control groups at different time points.

Variable	Group	On the day of discharge (Mean ± SD)	2 weeks after discharge (Mean ± SD)	1 month after discharge (Mean ± SD)	4 months after discharge (Mean ± SD)	Group Wald χ^2^ (*P*)	Time Wald χ^2^ (*P*)	Group × Time Wald χ^2^ (*P*)
Quality of life	Intervention	85.38 ± 12.46	100.43 ± 14.82	113.46 ± 11.05	119.29 ± 8.67	5.204 (0.023)	574.167 (< 0.001)	2.354 (0.308)
	Control	85.94 ± 10.42	98.14 ± 10.31	109.97 ± 10.25	116.82 ± 9.75			
	*t*	−0.353	1.306	2.515	1.945			
	*P*	0.725	0.193	0.013∗	0.053			
	Adjusted *P*	1.000	0.772	0.052	0.212			
	Mean difference (95% CI)	−0.56 (−3.65, 2.53)	2.29 (−1.15, 5.73)	3.49 (0.75, 6.23)	2.47 (−0.04, 4.98)			
Self-management efficacy	Intervention	90.27 ± 11.48	92.84 ± 13.14	99.67 ± 12.48	104.09 ± 11.92	6.573 (0.010)	301.390 (< 0.001)	3.971 (0.137)
	Control	89.14 ± 12.12	89.48 ± 12.99	94.56 ± 12.15	100.41 ± 12.31			
	*t*	0.697	1.864	3.024	2.214			
	*P*	0.487	0.064	0.003∗	0.028∗			
	Adjusted *P*	1.000	0.256	0.012∗	0.112			
	Mean difference (95% CI)	1.13 (−2.09, 4.35)	3.36 (−0.21, 6.93)	5.11 (1.72, 8.50)	3.68 (0.42, 6.94)			

SD, standard deviation; CI, confidence interval. The above data were corrected using the Benjamini-Hochberg procedure.

**Table 4:** Comparison of quality of life and self-management efficacy and their various dimensions after group intervention.

Variable	Group	On the day of discharge		2 weeks after discharge		1 month after discharge		4 months after discharge	
		Mean ± SD	*P*	Mean ± SD	*P*	Mean ± SD	*P*	Mean ± SD	*P*
Quality of life	Intervention	85.38 ± 12.46	0.725	100.43 ± 14.82	0.193	113.46 ± 11.05	0.013^a^	119.29 ± 8.67	0.053
	Control	85.94 ± 10.42		98.14 ± 10.31		109.97 ± 10.25		116.82 ± 9.75	
Physiological	Intervention	18.84 ± 4.85	0.803	20.48 ± 4.81	0.817	23.84 ± 3.16	0.103	24.74 ± 2.90	0.067
	Control	18.69 ± 4.03		20.34 ± 3.48		23.18 ± 2.73		24.00 ± 2.99	
Society	Intervention	18.66 ± 3.38	0.575	20.42 ± 5.20	0.186	22.53 ± 3.61	0.914	24.75 ± 2.99	0.180
	Control	18.94 ± 3.83		21.41 ± 5.65		22.58 ± 3.10		25.29 ± 2.81	
Emotion	Intervention	16.18 ± 3.86	0.422	17.55 ± 3.32	0.013^a^	18.67 ± 3.02	0.008^a^	19.30 ± 2.93	0.117
	Control	15.72 ± 4.45		16.48 ± 2.93		17.63 ± 2.59		18.63 ± 3.33	
Function	Intervention	10.19 ± 3.33	0.681	15.43 ± 3.52	0.042^a^	20.19 ± 3.76	0.087	21.37 ± 3.28	0.022^a^
	Control	10.39 ± 3.70		14.56 ± 2.56		19.41 ± 2.81		20.41 ± 2.81	
Additional attention	Intervention	21.51 ± 4.73	0.240	26.55 ± 4.43	0.027^a^	28.23 ± 3.37	0.012^a^	29.11 ± 3.64	0.226
	Control	20.20 ± 3.75		25.34 ± 3.44		26.99 ± 3.74		28.50 ± 3.75	
Self-management efficacy	Intervention	90.27 ± 11.48	0.487	92.84 ± 13.14	0.064	99.67 ± 12.48	0.003^a^	104.09 ± 11.92	0.028^a^
	Control	89.14 ± 12.12		89.48 ± 12.99		94.56 ± 12.15		100.41 ± 12.31	
Self-decompression	Intervention	29.89 ± 4.59	0.631	29.95 ± 5.68	0.570	34.99 ± 5.93	0.037^a^	36.66 ± 7.20	0.483
	Control	29.58 ± 4.59		29.50 ± 5.92		33.26 ± 6.08		35.97 ± 7.01	
Self-decision	Intervention	9.46 ± 2.08	0.665	11.56 ± 1.79	0.083	12.44 ± 1.94	0.010^a^	12.67 ± 2.13	0.427
	Control	9.32 ± 2.39		11.06 ± 2.32		11.70 ± 2.17		12.89 ± 1.94	
Positive attitude	Intervention	50.92 ± 6.72	0.452	51.30 ± 7.27	0.019^a^	52.24 ± 7.45	0.013^a^	54.76 ± 7.42	0.002^a^
	Control	50.23 ± 6.69		48.92 ± 7.46		49.60 ± 7.81		51.55 ± 7.65	

SD, standard deviation.

a *P* < 0.05.

**Table 5:** Paired comparison of estimated marginal means of quality of life and self-management efficacy.

Variable	Group	MD	Standard error	*t*	95% CI lower limit	95% CI upper limit
FACT-L	Control					
	T0-T1	−12.204	12.826	−9.988	−14.650	−9.757
	T0-T2	−23.852	12.178	−20.355	−26.175	−21.529
	T0-T3	−30.889	11.630	−27.601	−33.107	−28.670
	T1-T2	−11.648	6.527	−18.546	−12.893	−10.403
	T1-T3	−18.685	6.800	−28.557	−19.982	−17.388
	T2-T3	−7.037	4.478	−16.330	−7.891	−6.183
	Intervention					
	T0-T1	−15.048	15.198	−10.146	−17.989	−12.106
	T0-T2	−28.076	15.064	−19.098	−30.991	−25.161
	T0-T3	−33.905	14.174	−24.512	−36.648	−31.162
	T1-T2	−13.029	16.375	−8.153	−16.198	−9.860
	T1-T3	−18.857	16.471	−11.731	−22.045	−15.670
	T2-T3	−5.829	7.350	−8.125	−7.251	−4.406
SUPPH	Control					
	T0-T1	−0.343	6.590	−0.540	−1.600	0.914
	T0-T2	−5.426	7.053	−7.995	−6.771	−4.081
	T0-T3	−11.269	7.730	−15.150	−12.743	−9.794
	T1-T2	−5.083	7.724	−6.836	−6.557	−3.610
	T1-T3	−10.926	9.081	−12.504	−12.685	−9.194
	T2-T3	−5.843	6.716	−9.041	−7.124	−4.561
	Intervention					
	T0-T1	−2.552	6.265	−4.175	−3.765	−1.340
	T0-T2	−9.400	6.728	−14.317	−10.072	−8.089
	T0-T3	−13.819	8.224	−17.219	−15.411	−12.228
	T1-T2	−6.848	6.899	−10.171	−8.183	−5.513
	T1-T3	−11.267	9.840	−11.723	−13.171	−9.362
	T2-T3	−4.419	9.030	−5.015	−6.167	−2.672

MD, mean difference; CI, confidence interval; FACT-L, Functional Assessment of Cancer Therapy-Lung; SUPPH, Strategies Used by People to Promote Health.

### Effects on self-management

**Fig. 3:**
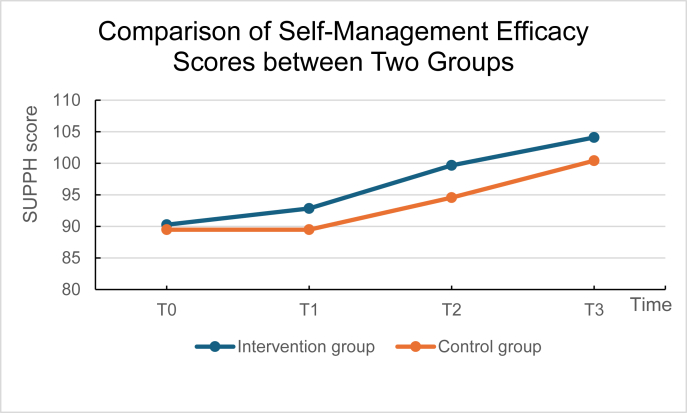
Changes in mean scores of self-management efficacy.

Analysis of self-management efficacy indicated significant main effects for group (Waldχ^2^ = 6.573, *P* = 0.010) and time (Waldχ^2^ = 301.390, *P* < 0.001), while the group × time interaction was not significant (*P* = 0.137). After adjustment for multiple comparisons, the intervention group showed significantly higher self-management efficacy scores than the control group at 1 month postoperatively (adjusted *P* = 0.012, mean difference = 5.11, 95% CI [1.72, 8.50]). In contrast, the between-group difference at 4 months did not remain statistically significant after correction (adjusted *P* = 0.112). No significant differences were detected between the two groups at baseline or at the 2-week follow-up. As shown in Fig. 3, the intervention group displayed a steeper increase in self-management efficacy scores from T0 to T3 relative to the control group. Additionally, both groups demonstrated accelerated improvement from T1 to T3 compared to the T0–T1 interval (Table 3, Table 4, Table 5 and Fig. 3).

## Discussion

### Main findings

To our knowledge, this study provides compelling evidence in China of a personalized follow-up program for postoperative lung cancer patients based on PROs. This program, utilizing a WeChat mini-program embedded with the MD Anderson Symptom Inventory and alarm thresholds, was led by nurses with multidisciplinary team support. It focuses on precise assessment and identification of personalized needs to deliver intelligent and accurate solutions. Over time, the two groups exhibited significant differences in quality of life and self-management efficacy. Notably, despite a small number of participants dropping out early in the study, all members of the intervention group actively engaged in the three follow-up assessments. Although their reporting frequency did not strictly adhere to all preset requirements, their sustained participation demonstrated high enthusiasm and compliance, thereby enhancing the study's reliability. Regarding the timepoint of the assessment, since the early recovery phase of post-operative lung cancer patients is a critical period for functional recovery, symptom management, and psychological adaptation, a 16-week improvement in self-management efficacy may indicate that patients are more likely to adhere to long-term rehabilitation plans (such as regular follow-ups and exercise), thereby indirectly influencing the 5-year survival rate. Studies have also emphasized that early post-operative symptom scores are associated with an increased risk of 5-year recurrence.

Contrary to our initial hypothesis, the generalized estimating equation analysis revealed no statistically significant group-by-time interaction effects for either quality of life or self-management efficacy. This indicates that the trajectory of improvement over time did not significantly differ between the intervention and control groups. However, the significant main effects of group and time suggest that both groups improved substantially over the study period, with the intervention group consistently demonstrating superior outcomes across multiple assessment points. The absence of a significant interaction effect may be attributed to several factors. First, the conventional follow-up care provided to the control group incorporated essential elements of post-surgical rehabilitation, resulting in considerable improvement. Second, the intervention's effect might manifest as a consistent between-group difference rather than a diverging trajectory over time. Finally, the study's statistical power to detect interaction effects in a model with multiple time points may have been limited. Notably, the between-group differences observed at specific time points suggest that the PRO-based follow-up program provided additional benefits beyond standard care, particularly during the critical early recovery phase.

The assessment of quality of life comprehensively captures the multidimensional aspects of patients' postoperative recovery, including functional restoration, subjective feelings, psychological status, and social activities, providing a detailed overview of postoperative rehabilitation.^5^ Denis et al.^21^ highlighted the potential of patient-reported data in predicting cancer recurrence and promoting early treatment. They also pioneered the idea that supportive care based on patient-reported data could effectively improve patients' quality of life. Although this benefit has not yet been directly demonstrated in their research, it opens new directions for future medical practices and care models. Our study aims to explore this hypothesis in depth. In our study, quality of life, as a main outcome measure, showed significant differences between the experimental and control groups in emotional and functional dimensions, as well as in additional patient attention. Regular symptom reporting through patient-reported tools allows health care teams to timely access this information,^34^ recommend specific diet, exercise, or psychological support programs, and provide early intervention for severe symptoms, thereby improving postoperative rehabilitation and quality of life.^35^^,^^36^ A recent systematic literature review^24^ emphasized that systematic evaluation of patient-reported data helps identify social and psychological issues, increasing the frequency of discussions on emotional function and the use of supportive therapeutic measures. However, our comparison of intergroup differences showed statistically significant differences in quality of life only at one month postoperatively. The observed effect attenuation may be attributed to early postoperative quality of life being predominantly influenced by surgical trauma and complications (e.g., infection, atelectasis), during which intervention efficacy could be counterbalanced by physiological recovery processes. Over time, reduced engagement with the digital platform may diminish perceived autonomy, weakening intervention effects. Concurrently, control-group patients likely approximated intervention outcomes through routine follow-ups or self-adjustments, particularly at 3–4 months post-surgery when physiological function typically stabilizes. During this phase, treatment-related adverse effects (e.g., chemotherapy/targeted therapy toxicity) and psychological adaptation challenges may offset initial intervention benefits. Prasongsook et al.^25^ also confirmed better trends in scores of living quality for patients using a lung cancer care application compared to routine care monitoring, but this was observed only in a small group of advanced lung cancer patients during the COVID-19 pandemic. Conversely, Kuo et al.^37^ found no significant improvement in patients' quality of life over a broader time frame. Although both studies were conducted in advanced lung cancer patients, their limited relevance to our results suggests that continuously improving patient quality of life is a complex, long-term process^21^ that future studies require consideration of various factors, including advances in medical technology, optimization of nursing services, and individual patient circumstances.

Self-efficacy, defined as an individual's confidence in their ability to perform specific behaviors, serves as a core element influencing patients' capacity for symptom self-management.^38^ It plays a pivotal role by shaping cognitive patterns, emotional responses, motivation, and behavioral performance.^39^ Enhancing patient self-efficacy represents not only an effective strategy for improving short-term emotional states but also a crucial mechanism for fostering long-term psychological adaptation and health outcomes. Our findings demonstrate that the personalized follow-up plan based on PROs significantly improved self-management efficacy in the intervention group. Although the absence of established minimal important difference values for this specific scale presents challenges in interpreting the clinical significance of the results, the observed improvement magnitude remains noteworthy. Compared with existing approaches, the superiority of our intervention lies in its functional comprehensiveness: a systematic review^40^ revealed that among existing electronic symptom reporting systems for cancer patients, fewer than half incorporated symptom self-management guidance, and less than one-third provided general educational information. In contrast, our follow-up system not only enables automated symptom monitoring and alert functions but also integrates structured health education, personalized rehabilitation guidance, and continuous feedback mechanisms.^41^ This systematic support enhanced patients' self-efficacy through multiple pathways: first, continuous symptom monitoring with immediate feedback enabled patients to accurately track their condition changes, strengthening their sense of control over disease management; second, tailored rehabilitation guidance helped patients acquire concrete, actionable self-management skills, thereby boosting their executive confidence; most importantly, the system established an ongoing patient-clinician communication mechanism, making patients feel sustained professional support - this sense of being accompanied and cared for itself constitutes a vital source of self-efficacy.^42^^,^^43^ Therefore, while further research is needed to establish definitive MID values, the multiple action mechanisms demonstrated by our intervention provide substantial support for its clinical value.

Analysis of barriers to self-management efficacy intervention revealed three major implementation challenges: First, disparities in digital literacy posed a significant barrier, with approximately 15% of elderly patients requiring additional telephone support due to technical difficulties with the WeChat mini-program. Second, adherence to symptom reporting demonstrated a declining trend over time, decreasing from 92% in the first month to 76% by the fourth month, particularly among patients with no or mild symptoms. Furthermore, the timeliness of multidisciplinary team responses was constrained by clinical workflow schedules, necessitating delayed processing until the next working day for symptom reports submitted outside regular hours.

### Implications for nursing practice and research

This study did not fully demonstrate the anticipated effects of the personalized follow-up program on the primary outcomes. The limited intervention efficacy appears attributable to several interrelated factors. First, successful implementation relied heavily on patients' proactive engagement and digital literacy. Despite standardized training, participation depth and benefits remained suboptimal among older or less digitally proficient patients, diluting overall effects. Second, intervention intensity was likely insufficient; the reactive “patient-report-triggered-response” model lacked proactive follow-ups and personalized guidance, potentially providing an inadequate intervention dose for clinically meaningful impact.

Furthermore, intervention effects naturally attenuated over time, consistent with patterns observed in digital health. As patients transitioned from acute recovery to stable phases, psychological reliance on the management tool diminished, compounded by waning novelty effects of digital interventions. Additionally, the usual care control condition involving telephone follow-ups provided substantial baseline support, potentially constraining the detectable between-group differences.

Building on these insights, future research should develop more dynamic and precise intervention models. Promising directions include implementing staged booster sessions at critical recovery timepoints, incorporating gamification and immediate feedback to sustain engagement, developing adaptive interventions tailored to early patient responses, and better integrating digital tools with in-person clinical services. These refinements may help overcome current limitations of remote health interventions and advance sustainable postoperative care models.

### Limitations

This study has several limitations. First, the use of a previously reported effect size for sample size estimation represents a potential methodological constraint. Although participants were recruited from a tertiary hospital in Shanghai—a national key specialty in thoracic surgery whose patient profile is representative of those undergoing lung cancer surgery in large specialized hospitals across China—the relatively small sample size may reduce statistical power and increase the risk of Type II error, thereby limiting robust inference regarding intervention effects. Future studies should aim to include participants from broader geographic regions and diverse backgrounds to provide higher levels of evidence. Second, due to constraints in clinical resources, the same group of nurses delivered both the experimental and control interventions. Although a series of measures were implemented to mitigate bias, this design still carries a risk of potential contamination between groups. Third, the outcome measures in this study were self-reported and thus may be subject to participant bias. In addition, the limited number of repeated assessments may have reduced the sensitivity to capture changes in PROs over time. Increasing the frequency of measurements in future studies could allow more detailed investigation of temporal trends. Fourthly, the heterogeneity in adjuvant treatment regimens—encompassing varying combinations and durations of chemotherapy, targeted therapy, and radiotherapy—may modulate the intervention's effectiveness. Our ongoing research agenda includes a focused evaluation of postoperative treatment as an effect modifier in digital health intervention outcomes. Finally, the enrolled sample had higher-than-average education levels and digital literacy. In addition, the availability of digital platforms such as WeChat varies across regions. This digital divide may limit the generalizability and international scalability of the intervention. To improve accessibility in underserved or older populations, future iterations could consider adaptive strategies such as: developing simplified user interfaces to reduce operational complexity; introducing caregiver co-access models to support patients with limited digital proficiency; and integrating voice assistant features to lower text-based interaction barriers. Such adaptations could help extend the reach of digital health interventions to broader patient populations.

## Conclusions

This study demonstrates that a 4-month patient-reported outcome-based follow-up program significantly improved postoperative self-management capabilities in lung cancer patients, confirming the short-term intervention effects. Although quality-of-life improvements were only significant at the 1-month postoperative mark, these findings still hold clinical value. However, a longer intervention period and continuous follow-up support may be needed to sustain these improvements in patient adherence and health outcomes. The satisfactory patient adherence observed in this study indicates the feasibility of integrating PROs into the continuity of care for postoperative lung cancer patients.

## CRediT authorship contribution statement

**Luo Yiqing:** Conceptualization, Methodology, Software. **Cheng Yuna:** Data curation, Writing - Original draft preparation. **Song Zuodong:** Visualization. **Chen Hui:** Writing - Reviewing and Editing. **Bo Yinping:** Supervision, Software, Validation. **ShiXing Haobo:** Investigation. All authors have read and approved the final manuscript.

## Ethics statement

The study was approved by the Institutional Review Board of the Shanghai Chest Hospital (Approval No. IS22099) and was conducted in accordance with the 1964 Helsinki Declaration and its later amendments or comparable ethical standards. All participants provided written informed consent.

## Data availability statement

The data that support the findings of this study are available on request from the corresponding author. The data are not publicly available due to the reason that the data containing information that could compromise the privacy of research participants.

## Declaration of generative AI and AI-assisted technologies in the writing process

No AI tools/services were used during the preparation of this work.

## Funding

No external funding.

## Declaration of competing interest

The authors declare no conflict of interest.
