# Supplementary material for: Patient-reported outcomes with a personalized follow-up program after lung cancer resection: A single-center randomized controlled trial
Source: Asia Pac J Oncol Nurs. 2026 Jan 2;13:100844. doi: 10.1016/j.apjon.2025.100844 (PMC12859223; doi:10.1016/j.apjon.2025.100844)
Supplement: Multimedia component 1 [file mmc1.doc]

**Appendix 2. Implementation strategies and specific contents of personalized follow-up program for lung cancer patients based on patient-reported outcomes.**

| **Implementation Strategies** | **Details** | **Contents** |
| --- | --- | --- |
| **Evidence transformation** | **——** | A systematic approach was undertaken to retrieve, evaluate, and synthesize the best available evidence on discharge preparation services for lung cancer surgery patients, establishing a foundation for clinical practice. **Methods:** Comprehensive searches were performed across international and domestic databases and resources including UpToDate, BMJ Best Practice, JBI, NICE, NGC, the Cochrane Library, PubMed, Yimaitong, China National Knowledge Infrastructure (CNKI), China Biomedical Literature Service System (CBM), Wanfang Database, and VIP Database. The search targeted guidelines, evidence summaries, best practice documents, clinical decision-support tools, systematic reviews, expert consensus statements, and randomized controlled trials (RCTs) pertaining to discharge continuity of care for lung cancer surgical patients. **Appraisal and Synthesis:** Identified literature was critically evaluated using standardized quality assessment tools, followed by evidence extraction, synthesis, and grading. **Outcome:** This process yielded 42 finalized recommendations across five core domains: pulmonary function rehabilitation exercises, physical training, nutrition and dietary guidance, psychological care, and symptom management. These recommendations constitute an evidence-based resource library for clinical reference by healthcare professionals. |
| **multidisciplinary collaboration** | **team building** | To ensure the effective implementation and operation of the intervention system, a dedicated, multidisciplinary team was established. The team is supervised by the department director and head nurse (the first author) and comprises 4 specialist nurses, 1 information technology (IT) specialist, 2 attending physicians, 1 nutritionist, 1 psychotherapist, and 2 rehabilitation therapists, forming an integrated postoperative management team. |
|  | **Division of labor** | - * Nurses: Responsible for the collection and initial evaluation of patient-reported symptom data, delivering structured health education and supportive care, and addressing patient inquiries through the online platform. - * Physicians: Provide expert guidance on disease progression, prognosis, and medication management. The IT specialist is accountable for software development, maintenance, ensuring data security and privacy, and troubleshooting technical issues encountered by users. - * Rehabilitation Therapists: Develop and oversee physical therapy and exercise rehabilitation programs, guiding patients in performing safe and appropriate activities. - * Psychotherapist: Assists patients in managing the emotional and psychological distress associated with their diagnosis and treatment, conducts psychological assessments and interventions, and provides coping strategies and counseling. - * Nutritionist: Assesses and addresses patients' nutritional requirements, formulates individualized dietary plans to enhance physical strength and immune function, and offers dietary strategies to manage treatment-related side effects. |
| **Team building and training** | **Basic and Specialized Application Training** | Training sessions were conducted in the department classroom. The first author delivered comprehensive instruction via slide presentations (three sessions total, each lasting 40 minutes) covering platform operation, patient-reported outcome (PRO) evaluation standards and parameter interpretation, and multidisciplinary collaboration protocols. The objectives were to ensure team proficiency in platform usage (including data entry and access), understanding the clinical significance of PROs, developing skills in data interpretation, and facilitating clinical decision-making based on PRO data. The responsibilities associated with each role and the collaborative workflow were clearly delineated, with emphasis placed on the critical importance of effective teamwork. |
|  | **Skills testing and mock exercise assessments** | - Conduct skills tests at the end of training to ensure each team member has mastered the necessary knowledge and skills. It includes 1 theoretical knowledge test and 2 practical operation assessments. - Evaluate the performance in simulation training, select 5 simulated patients for simulation evaluation, and provide feedback and improvement suggestions. Evaluation criteria include: response time, patient satisfaction, data accuracy and completeness, teamwork effectiveness, etc. |
|  | **Continuous quality improvement** | Based on the assessment results, we formulated improvement plans and conduct retraining on weak links. Working group meetings are held every Friday afternoon and team members are encouraged to submit improvement suggestions to the WeChat group at any time to continuously optimize workflow functions. |
| **Patient-Reported Outcomes Tool Settings—Application Ports and Functions** | **Permission settings** | After the medical staff log in for the first time, the engineer grants the attending doctor online consultation authority and the specialist nurse follow-up management authority based on the quality supervision and statistical authority granted to the chief physician and head nurse. |
|  | **patient terminal（Help patients complete registration in the mini program, import patient information into the personal file management module, and teach patients and primary caregivers how to use the software. and distribute operation brochures.）** | * Registration and Login: Patients register by providing basic demographic information (name, mobile number, identity card number, medical insurance card number) and subsequently log in using their mobile number and a password.  * Home Page: Features a personalized welcome message and provides navigation to core functional modules: Symptom Report, History, Reminders & Notifications, Health Assistant, Questionnaire Upload, and Personal Information.  * Symptom Reporting and Alert Thresholds: The standard reporting schedule entails twice-weekly submissions for the first month post-discharge, transitioning to once weekly until 4 months post-discharge (approximating 18 total reports), with provisions for ad-hoc reporting as needed. Patients complete the MD Anderson Symptom Inventory (MDASI), rating symptom severity and related distress on a numerical scale of 1-10. Scores of 4-5 activate a Yellow alert, while scores ≥6 activate a Red alert. A 'Submit' button finalizes report submission. An auto-save function preserves entered data if the session is interrupted.  * Historical Records: Patients can access a chronological record of their past symptom scores, presented alongside graphical trends and detailed historical reports.  * Reminders and Notifications: Automated reminder messages are delivered via WeChat at 14:00 on scheduled reporting days. If a report remains incomplete, subsequent reminders (displayed in yellow text) are sent after 1-hour and 2-hour intervals. Color-coded alerts inform patients when scores exceed predefined thresholds: red text indicates a high-severity symptom requiring attention, and green text confirms clinician acknowledgment of the report.  * Health Assistant: Disseminates time-sensitive discharge rehabilitation guidance and psychological support materials ("psychological station"). |
|  | **Medical staff terminal** | * Login: Access is secured via unique staff accounts and passwords.  * My Patients: Provides a searchable list of all registered patients, filterable by name or patient identification number.  * Patient Records: Comprises three sub-sections: 1) View Reports: Access the most recent and all historical symptom reports for individual patients; 2) Exception Report Alerts: System-generated notifications highlighting reports that surpass defined score thresholds; 3) Detailed Report: Enables viewing of a patient's detailed symptom scores and longitudinal trend graphs.  * Specialty Disease Management (Resource Library): Allows for the input of standardized response templates and the customization of personalized recommendations. The system also suggests potential interventions (e.g., scheduling follow-up appointments, adjusting treatment plans) based on reported symptom scores.  * Consultation Settings: Aggregates and summarizes symptom score data from all patients to generate statistical reports and analyze symptom patterns, aiding in the identification of high-risk individuals.  * Consultation Pool (Message Center): System Messages displays automatically generated system alerts; Patient Messages facilitates the viewing and management of direct patient communications. |
|  | **Information technology manager terminal** | * User Management: Provides dedicated interfaces for administering patient accounts and permissions, as well as staff accounts and access rights.  * Consultation Settings: Includes options for adjusting symptom score alert thresholds and for configuring notification rules and delivery methods.  * Data Backup and Recovery: Establishes routines for the regular backup of all patient data and critical system settings, enabling data restoration in the event of loss or corruption.  * System Maintenance: Oversees the maintenance, functional upgrades, and security management of the mini-program. Maintains comprehensive system operation logs to ensure security, performance monitoring, and operational traceability. |

**Specific Procedures and Timeline of the Intervention**

| Phase | Actor | Core Activities and Procedures |
| --- | --- | --- |
| Pre-discharge (Baseline) | Specialist Nurse | 1. Obtain written informed consent and complete baseline data collection.  2. Installation and Training: Assist the patient in installing the mini-program. Conduct a one-on-one operational training session (15-20 minutes) to ensure the patient can independently complete symptom reporting.  3. Manual Distribution: Provide the patient with a paper copy of the Patient Intervention Manual. |
| Intervention Period (Post-discharge, Months 1-4) | Patient | 1. Active Reporting: Complete the MDASI assessment via the mini-program according to the schedule (twice weekly in the first month post-discharge, then once weekly thereafter).  2. Receiving Reminders: Receive automatically pushed health education content from the system. |
|  | System | 1. Automated Alerting: Automatically generates alerts based on pre-set thresholds (MDASI single-item score: 4-5 = Yellow Alert; ≥6 = Red Alert).  2. Automated Reminders: Sends an automatic reminder at 14:00 on scheduled reporting days. If not completed, subsequent reminders are sent after 1 and 2 hours. |
|  | Specialist Nurse | 1. Daily Monitoring: Logs into the system on weekdays at 09:00 to review the "Abnormal Report Alert" list.  2. Standardized Response:  - Yellow Alert: Contacts the patient via phone or message within 24 hours for symptom assessment, provides first-level advice (e.g., breathing techniques, dietary adjustments) based on the Symptom Management Resource Library, and documents the response.  - Red Alert: Immediately (<4 hours) contacts the patient by phone for urgent assessment and simultaneously notifies the responsible Attending Physician via the system's internal messaging function. |
|  | Attending Physician | 1. Managing Red Alerts: Reviews the patient's situation within 24 hours of nurse notification to decide on the need for pharmacological intervention, an earlier outpatient appointment, or emergency care.  2. Online Consultation: Logs into the system at least three times weekly to respond to medically relevant questions from patients in the "Consultation Pool." |
|  | MDT | 1. Weekly Meeting: Holds a multidisciplinary team (MDT) meeting every Friday afternoon to review all alert cases, discuss complex situations, and optimize intervention strategies.  2. Consultation on Demand: Nurses or physicians can refer patients via the system's internal referral function to invite ad-hoc consultations from the nutrition, psychology, or rehabilitation teams based on patient needs. |
| **Study Endpoint (4 Months Postoperatively)** | **Specialist Nurse** | Conducts the final assessment of outcome measures and collects patient feedback on their satisfaction with the intervention process. |

**Manual for ePRO-Based Support Intervention Following Lung Cancer Surgery**

**1. Introduction**
This manual aims to provide all investigators involved in the "Study on ePRO-Based Support for Postoperative Lung Cancer Patients" with clear, standardized operating procedures. This ensures intervention consistency across all participants and guarantees the study's reproducibility.

**2. Standardized Operating Procedures (SOPs) for Team Members**

**2.1 SOP for Specialist Nurses**

- **2.1.1 Responsibilities:** Patient management, ePRO monitoring, first-line response, health education.
- **2.1.2 Stepwise Procedures:**
  - **Step 1: Patient Enrollment and Training (Pre-discharge)**
    1. Introduce the study to eligible patients using a standardized script.
    2. Obtain written informed consent.
    3. Guide patients through the registration and login process for the ePRO mini-program using a tablet computer.
    4. Conduct a "mock report": Guide the patient to complete the MDASI (M. D. Anderson Symptom Inventory) once in full, ensuring their understanding of each item's meaning and the scoring criteria.
    5. Distribute the paper-based Patient Guide, emphasizing the meaning of alert thresholds (Yellow/Red) and the corresponding actions to take.
  - **Step 2: Routine ePRO Monitoring and Response (Weekdays, 9:00 AM)**
    1. Log in to the healthcare staff terminal and access the "Abnormal Report Alert" module.
    2. **Managing Yellow Alerts:**
       - Locate the patient in the list and click to access the "Detailed Report".
       - Document the symptomatic item(s), score(s), and trend(s).
       - Place a standardized follow-up phone call within 24 hours.
       - **Phone Script Template:** "Hello, [Patient Name]. This is your specialist nurse, [Nurse Name]. We noted your report from yesterday indicated a score of [Score] for [Specific Symptom, e.g., 'pain']. We would like to understand more about this symptom – could you describe its specific characteristics and duration?..." Provide advice based on the Symptom Management Resource Library and record the interaction in the system's "Consultation Pool".
    3. **Managing Red Alerts:**
       - Place an urgent assessment phone call immediately (<4 hours).
       - **Phone Script Template:** "Hello, [Patient Name], we see your reported [Symptom] score is very high and we are very concerned about your current condition..." Quickly assess the severity of the symptom and the presence of any emergent risks.
       - Following assessment, immediately notify the responsible attending physician via the system's internal messaging function using the message template: "【RED ALERT】Patient [Name], ID[XXX], reported [Symptom] score [XX]. Preliminary phone assessment completed. Summary: [...]. Please review promptly."
  - **Step 3: Health Education Delivery**
    - According to the schedule published in the system's "Health Assistant" module, use standardized materials to provide guidance on relevant topics (e.g., pulmonary function exercises, nutrition) during patient follow-up visits or telephone consultations.

**2.2 SOP for Attending Physicians**

- **2.2.1 Responsibilities:** Medical decision-making, review of red alerts, online consultation.
- **2.2.2 Stepwise Procedures:**
  - **Step 1: Review of Red Alerts (Within 24 hours of notification)**
    1. Log in to the system to review the nurse's notes and the patient's detailed report.
    2. Contact the patient directly by phone if necessary for a more in-depth medical assessment.
    3. Make a clinical decision and document it in the system: a) Continue observation; b) Prescribe medication; c) Recommend an earlier outpatient follow-up; d) Recommend visiting the emergency department.
  - **Step 2: Online Consultation (At least three times per week)**
    1. Access the "Consultation Pool" to review patient messages.
    2. Provide a professional and clear response within 48 hours. For non-medical issues (e.g., psychological, nutritional), utilize the system's referral function to direct the query to the appropriate specialist.

**3. Symptom Management Resource Library (Excerpt)**

**3.1 Symptom: Pain (MDASI Score ≥ 4)**

- **3.1.1 First-Level Intervention (Performed by Nurse):** Assess pain location and character. Guide the patient in non-pharmacological techniques (e.g., relaxation breathing, distraction). Confirm compliance with prescribed discharge medications (non-opioid).
- **3.1.2 Second-Level Intervention (Trigger: Score ≥6 or failure of first-level intervention):** The nurse escalates to the physician. Following assessment, the physician considers adjusting the analgesic regimen (e.g., adding a weak opioid).

**3.2 Symptom: Dyspnea (MDASI Score ≥ 4)**

- **3.2.1 First-Level Intervention:** Instruct the patient to immediately employ energy conservation techniques (e.g., resting between activities) and pursed-lip breathing.
- **3.2.2 Second-Level Intervention:** Escalate to the physician for assessment regarding potential oxygen therapy or prescription of bronchodilators.

**4. Intervention Fidelity and Quality Assurance**

- **4.1 Weekly MDT Meetings:** The fixed agenda includes: reviewing all alerts and handling records from the previous week; discussing cases of suboptimal management; collective review of specific sections of this Intervention Manual.
- **4.2 Skills Recertification:** Conduct simulated assessments of ePRO system operation and response procedures for team members quarterly.
- **4.3 Data Monitoring:** The project coordinator regularly reviews system logs to ensure compliance with key performance indicators, such as response times (Yellow <24h, Red <4h).
